# Active season body mass patterns of little brown and northern myotis bats

**DOI:** 10.1002/ece3.9230

**Published:** 2022-08-22

**Authors:** Evan W. Balzer, Adam D. Grottoli, Lynne E. Burns, Hugh G. Broders

**Affiliations:** ^1^ Department of Biology University of Waterloo Waterloo Ontario Canada; ^2^ Environment and Climate Change Canada Edmonton Alberta Canada

**Keywords:** bats, foraging, hibernation, little brown myotis, mass, northern myotis

## Abstract

Animals are expected to adjust their behavioral patterns to improve fitness outcomes, such as fecundity or offspring survival. For long‐lived hibernators, decisions made in each annual cycle may reflect considerations not just for concurrent survival and reproduction but also the pressure to maximize overwinter survival and future reproductive success. We examined how these elements manifest themselves in the body mass variation patterns of North American northern latitude temperate bats, whose size and roosting habits present considerable monitoring challenges. We characterized and compared the summer and fall mass variation patterns of little brown myotis (*Myotis lucifugus*) and northern myotis (*M. septentrionalis*) from a historic dataset. In summer, the estimated date of parturition was strongly associated with spring foraging conditions (low wind, low precipitation, and warm temperatures), and mass gain associated with female reproduction conferred considerable differentiation between the mass variation patterns of females and males. In fall, differences were most apparent among species, although adults exhibited a greater capacity for rapid mass gain than juveniles. These results demonstrate how reproductive constraints and interannual survival have important influences on the behavior of temperate bats. Future work should seek to quantify the fitness benefits of patterns identified in this study, such as the rate of prehibernation mass gain.

## Graphical abstract

Temperate hibernating bats are constrained by energetic tradeoffs associated with hibernation and reproduction. We identified sex‐associated differences in summer mass variation and characterized an age‐associated difference in the capacity for prehibernation mass gain in little brown (*Myotis lucifugus*) and northern myotis (*M. septentrionalis*). We also identified a positive relationship between relatively good foraging conditions and early parturition in females.

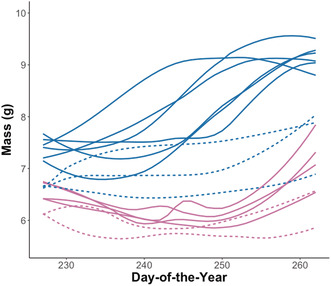

## 1 INTRODUCTION

Through selective pressure, populations evolve behavioral strategies to maximize fitness (Stearns, 1976). Because behavior happens in the context of environmental conditions, these strategies must be interpreted in light of abiotic and biotic influences (e.g., Burles et al., 2009) and the behavioral or phenotypic flexibility they elicit in animals (Pigliucci, 2001; Snell‐Rood, 2013). These nuances may be especially evident in seasonal environments where annual weather patterns are characterized by distinct periods of temperature and precipitation intensity (Bonan, 2002; Sunday et al., 2011). For example, many bird species spend the summer at breeding grounds in the northern part of their range and migrate toward the tropics during the winter (Newton & Dale, 1996). Conversely, some mammals remain near their summer territories and hibernate through winter (e.g., Grizzly Bears; Nelson et al., 1983).

Patterns of behavior in North American temperate hibernating bats, such as little brown myotis (*Myotis lucifugus*) and northern myotis (*M. septentrionalis*), are notably influenced by seasonality. These long‐lived insectivorous mammals give birth during spring and summer (Wimsatt, 1960) and use prolonged torpor to hibernate through the winter when food is scarce (Caceres & Barclay, 2000; Czenze & Willis, 2015; Fenton & Barclay, 1980; Whitaker & Gummer, 1992). During hibernation, individuals suppress their metabolic rate and subsist on fat stores acquired in the preceding active season (Carey et al., 2003; Geiser, 2004; Thomas et al., 1990). The demands of hibernation illustrate the necessity of not only physiological adaptations associated with long‐term torpor but behavioral ones as well. Indeed, these behavioral adaptations have important implications for inter‐annual survival (Frick, Reynolds, et al., 2010; Norquay & Willis, 2014), and place special emphasis within these species on the ability to access and capitalize on food resources, especially for endangered species affected by white‐nose syndrome (WNS), an emergent disease of hibernating bats (Blehert et al., 2009; Cheng et al., 2021; Environment and Climate Change Canada, 2018; Frick, Pollock, et al., 2010).

Collectively, behavioral adaptations can be considered according to both the rate of mass change and the timing of mass maxima, minima, and onsets of change. In fall, northern latitude temperate bats of all age classes and sexes maximize their net energy budgets to gain fat and survive hibernation. During this period, individuals may experience changes in body mass as dramatic as 38% (Kronfeld‐Schor et al., 2000) with rates of mass gain as high as 0.1 g/day (Kunz et al., 1998). Throughout the winter, periodic arousals from hibernation drive an expenditure of fat mass to a collective loss of around 25% (Fenton, 1970; Thomas et al., 1990). Because these arousals are energetically intensive, an increased arousal frequency is likely a driving factor for WNS mortality (Lilley et al., 2016; Reeder et al., 2012). The annual cycle is thus generally characterized by a peak body mass prior to hibernation, a long period of gradual mass loss during hibernation, and then a resumption of activity and mass gain upon emergence in spring.

Within this cycle, nuances among the mass variation patterns of some North American temperate bats may be explained by several key factors. The first factor to consider is sex, which has a major influence on the reproductive investment of adults (Fenton, 1969; Kunz et al., 1998; Schowalter, 1980). Adult males undergo relatively inexpensive spermatogenesis throughout the summer and subsequently copulate promiscuously at swarming sites in the fall (Norquay & Willis, 2014; Schowalter, 1980; Thomas et al., 1979). This late annual investment may to some degree explain why males emerge from hibernation later than females (Czenze & Willis, 2015) and persist in areas with relatively low prey abundance during the summer (Barclay, 1991). The comparatively larger female investment takes place in spring and early summer (Wimsatt, 1960), and the Thrifty Female Hypothesis suggests females should give birth as soon as possible each year to maximize overwinter survival of offspring (Czenze et al., 2017; Jonasson & Willis, 2011; Norquay & Willis, 2014). However, the optimal time of parturition will vary with past and present environmental conditions (Linton & Macdonald, 2020), geographic region (Rodrigues et al., 2003), and individual characteristics such as body condition or reproductive experience (Linton & Macdonald, 2019).

Another potential contributor to mass variation pattern differences is individual age. Unlike adults, juveniles are generally not reproductively active during fall swarming (Thomas et al., 1979) which may allow them to devote more effort to foraging. Considering high juvenile overwinter mortality (Frick, Reynolds, et al., 2010), there is an incentive for juveniles to forgo reproductive effort if energy sequestering capacity is limiting. Conversely, adults are reproductively active at swarming sites (Thomas et al., 1979), which may necessitate a tradeoff between reproductive activity and fat deposition. Since mass gain can be achieved through either hyperphagia (Kronfeld‐Schor et al., 2000; McGuire et al., 2009) or increased torpor use (McGuire et al., 2016; Speakman & Rowland, 1999; Stawski et al., 2014), differences in foraging efficiency (McGuire et al., 2009) between adults and juveniles may further dictate when and how prehibernation mass gain takes place.

The third influence on mass variation is that of foraging conditions. For volant insectivorous species like little brown myotis and northern myotis, energy intake varies with insect availability, ambient temperature, wind speed, and precipitation, such that foraging efficiency is diminished on nights with lower temperatures and high winds and/or precipitation (Anthony et al., 1981; Ciechanowski et al., 2007; Fenton, 1969). High precipitation in temperate areas is furthermore associated with delayed parturition (Grindal et al., 1992; Linton & Macdonald, 2018, 2020) and reduced swarming site activity in fall (Parsons et al., 2003). Just as these factors affect energy intake in bats, they may also affect energy expenditures. In particular, low ambient temperatures increase the costs of euthermia and explain, at least in part, torpor use throughout the year (Besler & Broders, 2019; Dzal & Brigham, 2013; Speakman & Rowland, 1999; Willis et al., 2006). Relatively early female spring emergence (Czenze & Willis, 2015; Davis & Hitchcock, 1965; Norquay & Willis, 2014) often coincides with limited insect abundance and unfavorable environmental conditions (e.g., low temperature, precipitation, and wind) which necessitate torpor use to conserve energy and recover from hibernation (Besler & Broders, 2019; Czenze & Willis, 2015; Humphries et al., 2003). However, because torpor use delays parturition and weaning (Racey & Swift, 1981), poor spring foraging conditions may in turn delay parturition such that reproductive females and their offspring struggle to gain sufficient prehibernation energy stores. With WNS, insufficient energy stores become an even greater issue since bats entering hibernation with larger fat stores appear to have a greater chance of surviving overwinter WNS infection (Cheng et al., 2019). As climate change alters the phenology of hibernating and prey species (Forrest, 2016; Lane et al., 2012) and introduced pathogens impact hibernating bats (Blehert et al., 2009), it is increasingly important to develop quantitative ways to assess patterns of annual phenology within and among species, and especially those whose phenology is closely linked with environmental conditions.

Given the magnitude of reproductive and prehibernation investments in temperate bats, disparate behavioral strategies among sexes, age classes, and species should confer detectable and distinct annual patterns of body mass change. Unfortunately, contemporary body mass comparisons were often limited to paired mean comparisons at points throughout a year (e.g., Kunz et al., 1998; Rughetti & Toffoli, 2014) which may fail to capture meaningful variation across seasons. Available records of mass variation patterns of little brown myotis and northern myotis (Fenton, 1970; Kronfeld‐Schor et al., 2000; Kunz et al., 1998; Lacki et al., 2015; McGuire et al., 2009, 2016; Schowalter, 1980; Speakman & Rowland, 1999; Townsend et al., 2008) have identified some regional characteristics of body mass variation in these species, but we sought to expand on this work through more comprehensive inter‐year comparisons.

The goal of this project was to characterize the active season mass variation patterns of little brown myotis and northern myotis. To achieve this goal, we set three objectives. First, we evaluated summer mass variation patterns in adults to identify how their form, magnitude, and variation differed according to sex and species. Second, we similarly examined patterns of mass variation in fall but included juveniles to determine whether they displayed a similar capacity to gain mass as adults and whether such gain took place at a similar time. Our last objective was to identify whether the temporal distribution of summer mass variation in females could be explained by weather conditions in either spring or summer.

It should be noted that Pd has caused WNS and the devastation of populations of local species within our study area, which are now listed as “endangered” under provincial and federal legislation. Although our work does not test or add directly to our understanding of the impacts of WNS, it does provide some understanding of the inter‐year‐, ‐specific, ‐sex, and ‐age variation patterns of mass gain. Cheng et al. (2019) found that bats with higher early‐winter fat stores have a higher probability of overwinter survival in the face of WNS and we aim to provide insight into the vulnerability of different groups of bats to WNS‐associated mortality.

## 2 METHODS

### 2.1 Sample selection

To achieve these objectives, we selected data from projects conducted in the Canadian provinces of New Brunswick, Nova Scotia, Prince Edward Island, Ontario, and the island of Newfoundland between 1999 and 2019. In these projects, bats were captured with mist nets (Avinet) and harp traps (Austbat Research Equipment) by different research groups and assessed for standard morphometric and diagnostic criteria, including sex, age class (adult or juvenile; Kunz & Anthony, 1982), and mass (g) to two decimal places. Upon initial screening of the dataset, we chose to proceed with little brown myotis and northern myotis because they were the two species with sufficient data for an informative comparison. The capture dates were recorded as Julian Date (1–365), and individual observations were grouped for analysis according to year, province, age class (adult/juvenile), sex, and species (e.g., adult male *M. lucifugus* captured in Nova Scotia in 2013; hereafter “group”). To exclude swarming behavior in summer analyses, we classified summer captures as those that occurred before August 11 (approximately Julian Day 222) (Burns & Broders, 2015) at locations other than known or suspected hibernacula. We used this cutoff date because it represents a conservative estimate of the onset of swarming behavior in the region (Burns & Broders, 2015). We made no temporal constraint for the inclusion of juvenile records in fall because it was assumed that the importance of surviving their first winter should orient all juvenile fall behavior toward the mass gain necessary for overwinter survival. Adult records were only included for fall analysis if they occurred at known or suspected hibernacula and swarming sites. We chose a location criterion for adults because the migration to such sites was assumed to be a choice to engage in a new suite of behaviors. Furthermore, monitoring at such sites in Nova Scotia suggests very low summer occupancy until the swarming period in fall (Burns and Broders, unpublished data).

For summer, we selected groups representing each of the possible adult sex/species combinations (e.g., adult male *M. lucifugus*; hereafter “collection”) that had the best sampling regime (i.e., based on the temporal distribution and quantity of sampling). Groups and collections are distinguished in this analysis by their specificity; collections are more broad and, unlike groups, do not differentiate data according to either location or year. Each chosen group included at least nine unique nights of sampling with no sampling gaps longer than 20 days so that biologically relevant phenomena (e.g., pregnancy) would be detectable even when sampling regimes were irregular (Chen et al., 2002; Lepot et al., 2017). Similarly, we selected fall data from capture efforts according to the same criteria, for 15 groups, including adults and juveniles of both sexes in three consecutive years (2009–2011).

### 2.2 Time series comparison

Given these groups, we fitted LOESS (Cleveland, 1979; Cleveland & Devlin, 1988) nonparametric fits (span = 0.75) through the full sample of body mass values of each group and trimmed each fit to the widest possible range for which captures were available in all groups; Julian dates 158–207 (approximately June 7–July 26) for summer and 227–262 (approximately August 15–September 19) for fall. We chose not to fit exclusively between these dates because doing so would lose accuracy provided by captures outside that range in years with more sampling. We chose a quadratic fit instead of a linear fit because quadratic smoothing is preferable when the data include informative curvature (Cleveland & Devlin, 1988). Similarly, we selected a span value of 0.75 because our interest was primarily in identifying large‐scale trends rather than daily fluctuations, thus necessitating a wider evaluation window (Cleveland & Devlin, 1988).

To test whether differences among group mass variation patterns could be explained on the basis of sex or species, we first constructed a dynamic time warp (DTW; Berndt & Clifford, 1994; Sakoe & Chiba, 1971) dissimilarity matrix among the time series of each group with the R package *dtwclust* (Sardá‐Espinosa, 2019). Dynamic time warping is a time series comparison technique that determines the optimal nonlinear alignment between pairs of time series that minimizes the sum of absolute differences between the two series (Rabiner et al., 1978). In DTW analysis, the order of points in time series is retained, but their distribution through time is nonlinearly “warped” such that each point is paired with one or more points in a comparison sequence (Berndt & Clifford, 1994). Dissimilarity between two or more series is therefore evaluated according to their features, regardless of whether they have the same onset, duration, or amplitude (Aghabozorgi et al., 2015; Sakoe & Chiba, 1971). One common application is in speech analysis, in which shared phrases can be identified among speakers, despite differences in volume or pace of speech (Amin & Mahmood, 2008). In ecology, this technique may also be used to generate a dissimilarity matrix among annual time series to determine whether sampled populations share biologically significant features, regardless of when those features appear in the sampling period. Given a DTW dissimilarity matrix, we then performed a Ward's distance unsupervised hierarchical clustering exercise (Ward, 1963) and organized the outcome as a dendrogram.

To facilitate comparison among groups, we made inferences based on the characteristics of each fitted line. In both summer and fall, we calculated the first derivatives of each fitted line as an estimate of the intensity, direction, and variability of mass change in each group. In summer, we also identified the earliest and latest fitted value as a metric of early and late season variation among groups. Because the fitted period of fall sampling was comparatively short and we were primarily interested in how bats prepare for hibernation, we instead estimated the greatest magnitude in mass change within each group's fitted window. We also examined fall fitted lines for evidence of sudden onset in rapid mass gain. In cases where a clear onset was not evident, we estimated the second derivate of mass change in fall, whose greatest values indicated when the rate of mass gain changed the most and thus, estimated when the onset of the rapid mass gain occurred.

### 2.3 Foraging conditions

To test for the influence of foraging conditions on the timing of parturition, we selected all summer female groups from the historical dataset with available local environmental data (*n* = 9) according to the same eligibility criteria as previous and fitted them in the same way. Given the resulting fitted lines, we selected the highest fitted mass value as an estimate of the group mean parturition date. We collated environmental data for each of these groups, which consisted of the hourly temperature, hourly wind speed (m/s), and hourly precipitation (mm) for each sampling location and were taken from the nearest Environment and Climate Change Canada weather station that logs hourly data. Using these variables, a foraging index, created by Linton and Macdonald (2018), was used to assess the hourly foraging conditions from April to July. A score of 0 (temp. <7°C; wind >5 m/s; rain >0.75 mm), 0.5 (7°C ≤ temp. ≤ 10°C; 4 m/s ≤ wind ≤ 5 m/s; 0.25 mm ≤ rain ≤ 0.75 mm), or 1 (temp. >10°C; wind <4 m/s; rain <0.25 mm) was assigned to each hour between sunset and sunrise (determined using *SunCalc*; Thieurmel & Elmarhraoui, 2019) to represent poor, moderate, and good foraging conditions, respectively. A spring suitable foraging condition (SpSFC; April and May) and summer suitable foraging condition (SuSFC; June and July) were then calculated for each cohort using the arithmetic average of the hourly foraging condition (Linton & Macdonald, 2018). In groups whose parturition inflection dates were estimated from bats captured at multiple locations, we used a weighted average in which the respective weight of data collected from a particular station was equal to the proportion of bats caught at that location relative to the rest of the cohort. After testing for normality, we tested for a correlation between the estimated parturition date and SpSFC and SuSFC each with linear regression.

## 3 RESULTS

**FIGURE 1:**
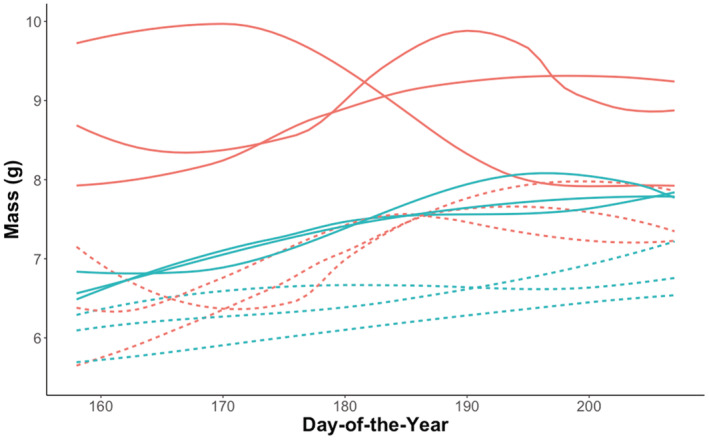
Fitted summer mass variation patterns of adult little brown myotis (*Myotis lucifugus*; solid lines) and northern myotis (*M. septentrionalis*; dashed lines) captured between Julian days 158 and 207. Female groups are denoted in orange, and male groups are denoted in aqua.

**FIGURE 2:**
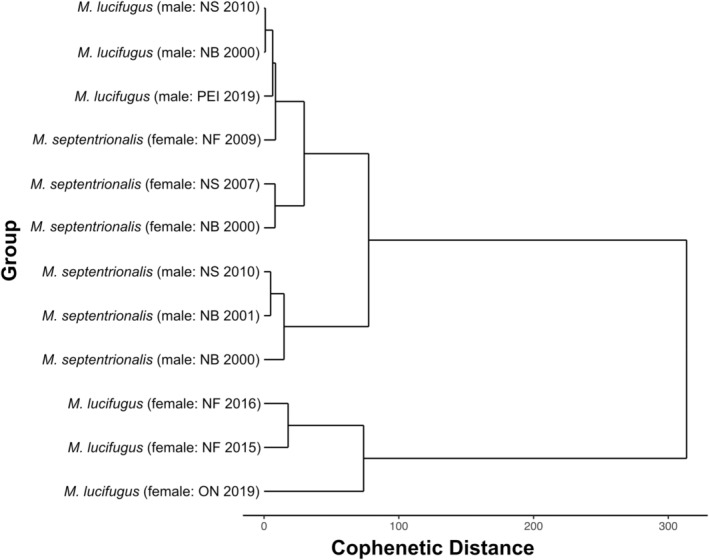
Hierarchical dynamic time warp distance clustering dendrogram of time series constructed from summer mass variation patterns of adult little brown myotis (*Myotis lucifugus*) and northern myotis (*M. septentrionalis*) captured in New Brunswick (NB), Nova Scotia (NS), Prince Edward Island (PEI), Newfoundland (NF), and Ontario (ON) between 2000 and 2019. The distance among clusters is cophenetic, which indicates the point at which a pair of adjoined clusters may be combined.

**FIGURE 3:**
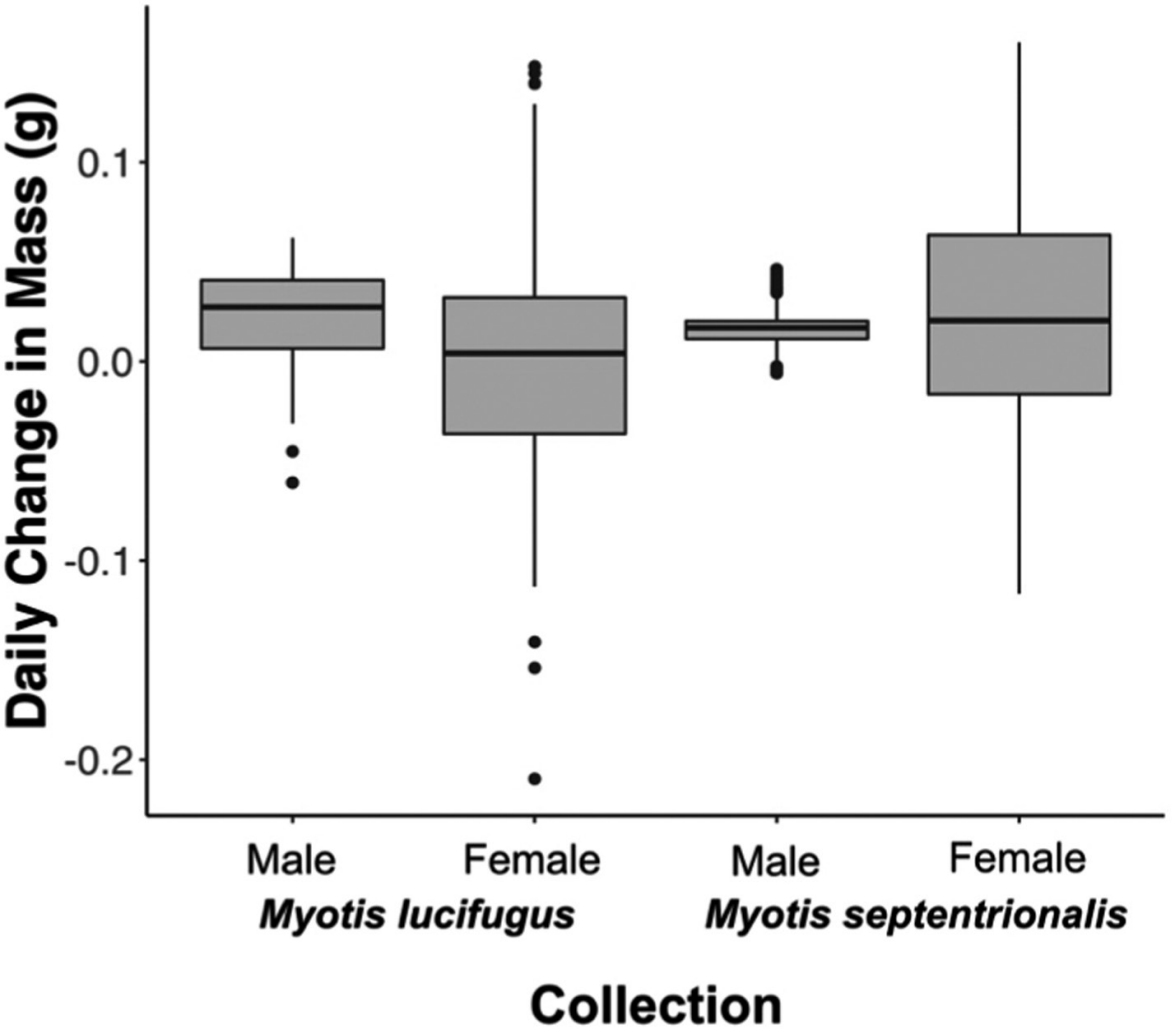
Distribution of the summer daily body mass change in collections of adult male and female little brown myotis (*Myotis lucifugus*) and northern myotis (*M. septentrionalis*) captured in Eastern Canada between 2000 and 2019. Each boxplot is comprised of 49 points, each representing the estimated daily change in body mass for the group between Julian dates 158 and 207 (approximately June 7–July 26). Error bars represent the standard error of the estimated mean daily change in body mass.

The 12 summer groups (Figure 1; Tables 1 and 2) were assorted into four DTW clusters, which were statistically distinguishable by species and sex (Figure 2). The only exception to this assortment was the group of female northern myotis captured in 2009 in Newfoundland, which was most similar to the male little brown myotis cluster. Of all the clusters, female little brown myotis were the most differentiated from the others (cophenetic distance = 313.57), including female northern myotis (cophenetic distance = 429.11). Male groups of both species demonstrated steady rates of mass increase throughout the summer, but the rate of change in female groups was much more variable (Levene's test: *p* < .001 for both species) (Figure 3).

**TABLE 1:** Sampling characteristics of groups of adult little brown myotis (*Myotis lucifugus*) and northern myotis (*M. septentrionalis*) captured in New Brunswick (NB), Nova Scotia (NS), Prince Edward Island (PEI), Newfoundland (NF), and Ontario (ON) during the summer between 2000 and 2019.

Species	Sex	Year	Province	Earliest capture	Latest capture	Largest gap	*n*	Nights	Sites
*M. lucifugus*	Male	2000	NB	151	222	15	27	14	1
*M. lucifugus*	Male	2010	NS	122	209	16	124	21	7
*M. lucifugus*	Male	2019	PEI	158	221	15	22	12	5
*M. lucifugus*	Female	2015	NF	139	220	9	294	31	2
*M. lucifugus*	Female	2016	NF	154	216	9	153	19	2
*M. lucifugus*	Female	2019	ON	134	219	11	298	21	6
M. lucifugus*	Female	2012	NS	147	214	17	142	12	9
M. lucifugus*	Female	2013	NF	158	214	18	616	26	2
*M. septentrionalis*	Male	2000	NB	142	222	13	37	18	1
*M. septentrionalis*	Male	2001	NB	156	207	17	20	9	1
*M. septentrionalis*	Male	2010	NS	120	209	17	31	15	4
*M. septentrionalis*	Female	2000	NB	142	222	9	30	17	1
*M. septentrionalis*	Female	2007	NS	149	209	9	29	14	2
*M. septentrionalis*	Female	2009	NF	156	217	12	28	14	2
M. septentrionalis*	Female	2005	NS	154	216	7	62	20	2
M. septentrionalis*	Female	2009	NF	156	217	18	28	14	2

*Note*: Earliest capture and latest capture denote the date (Julian date) of the first and last capture in each group, and the largest gap in sampling within those dates is largest gap. The sample size, number of unique capture nights, and total number of capture sites are given as *n*, nights, and sites respectively. Groups added for parturition analysis are indicated with *.

**TABLE 2:** Summary of the LOESS‐fitted interpolations of mass values of groups of adult little brown myotis (*Myotis lucifugus*) and northern myotis (*M. septentrionalis*) captured during the summer in New Brunswick (NB), Nova Scotia (NS), Prince Edward Island (PEI), Newfoundland (NF), and Ontario (ON) between 2000 and 2019.

Species	Sex	Year	Province	*SE*	Earliest mass (g)	Latest mass (g)	Change (g)
*M. lucifugus*	Male	2000	NB	0.59	6.49	7.84	1.35
*M. lucifugus*	Male	2010	NS	0.50	6.56	7.79	1.22
*M. lucifugus*	Male	2019	PEI	0.60	6.84	7.77	0.94
*M. lucifugus*	Female	2015	NF	1.10	7.93	9.24	1.31
*M. lucifugus*	Female	2016	NF	1.27	8.69	8.88	0.19
*M. lucifugus*	Female	2019	ON	1.03	9.72	7.92	−1.80
M. lucifugus*	Female	2012	NS	0.77	8.16	8.43	−0.97
M. lucifugus*	Female	2013	NF	0.84	7.39	7.19	1.03
*M. septentrionalis*	Male	2000	NB	0.52	5.69	6.54	0.85
*M. septentrionalis*	Male	2001	NB	0.82	6.29	6.76	0.47
*M. septentrionalis*	Male	2010	NS	0.68	6.09	7.22	1.12
*M. septentrionalis*	Female	2000	NB	0.65	5.65	7.35	1.70
*M. septentrionalis*	Female	2007	NS	0.67	6.38	7.23	0.85
*M. septentrionalis*	Female	2009	NF	1.46	7.15	7.86	0.71
M. septentrionalis*	Female	2005	NS	0.91	6.61	7.02	0.39
M. septentrionalis*	Female	2009	NF	1.46	7.15	7.86	0.71

*Note*: Earliest mass and latest mass describe the fitted mass value on days of year 158 and 207, respectively, and change denotes the net difference in mass between those values. Standard error (*SE*) describes the error of each full LOESS fit (span = 0.75), including all values between the earliest available capture until the latest available capture, up to day‐of‐year 222. Groups added for parturition analysis are indicated with *.

**FIGURE 4:**
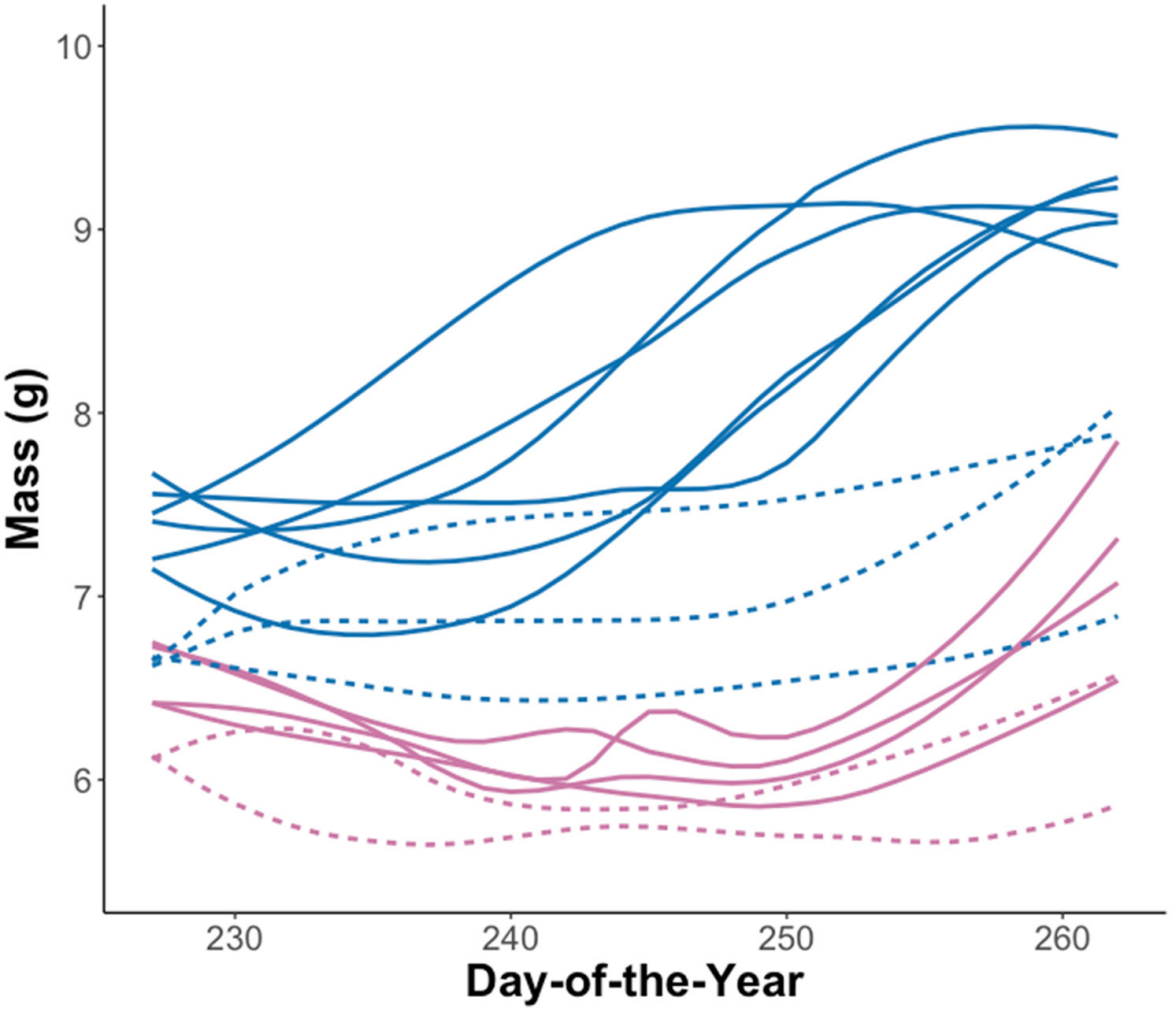
Fitted fall mass variation patterns of little brown myotis (*Myotis lucifugu*s; blue) and northern myotis (*M. septentrionalis*; purple) captured in Nova Scotia between Julian dates 227 and 262. Adult groups are denoted with solid lines and juvenile groups are denoted with dashed lines.

**FIGURE 5:**
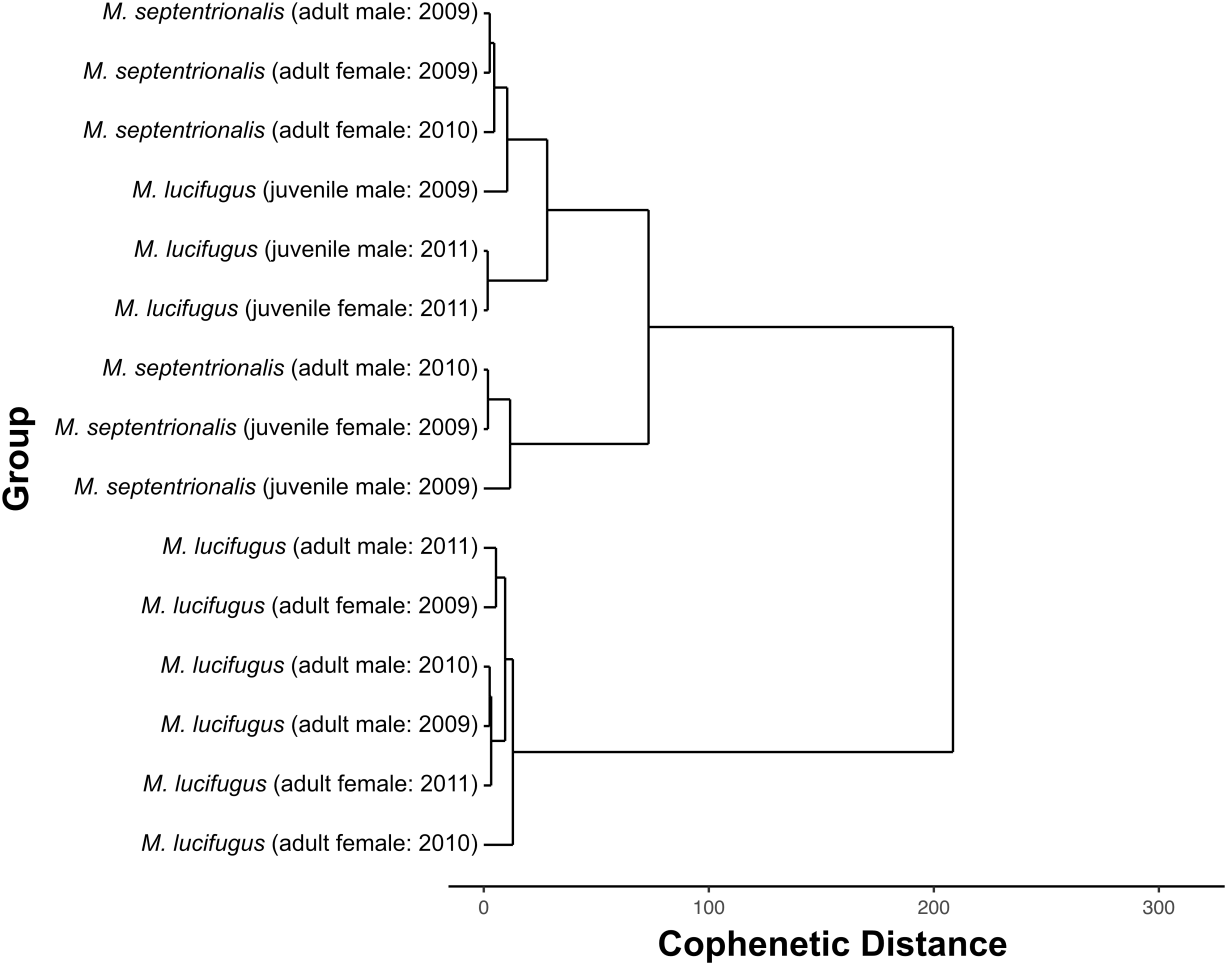
Hierarchical dynamic time warp distance clustering dendrogram of time series constructed from fall mass variation patterns of little brown myotis (*Myotis lucifugus*) and northern myotis (*M. septentrionalis*) captured in Nova Scotia between 2009 and 2011. The distance among clusters is cophenetic, which indicates the point at which a pair of adjoined clusters may be combined.

**FIGURE 6:**
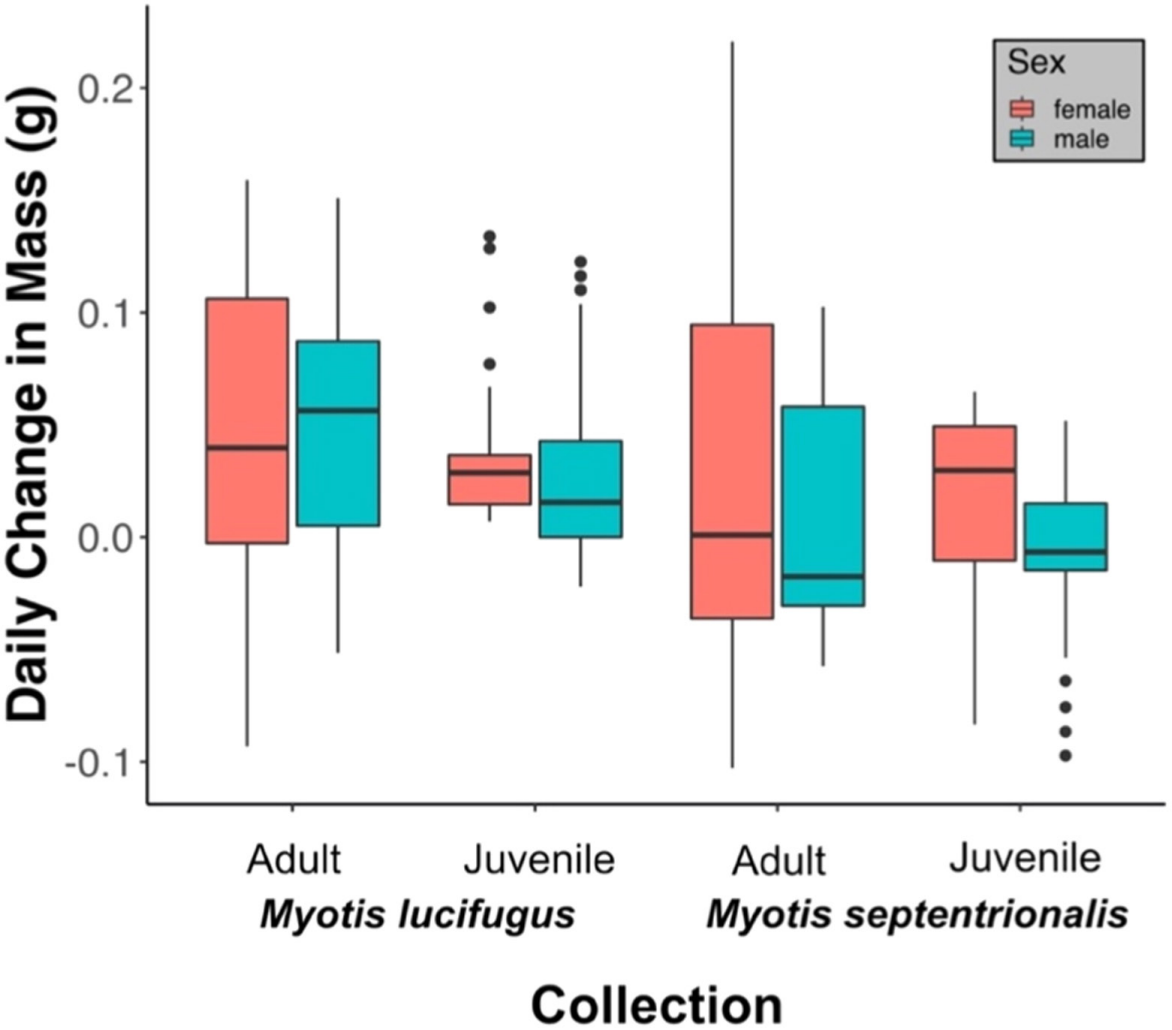
Distribution of the fall daily body mass change in collections of little brown myotis (*Myotis lucifugus*) and northern myotis (*M. septentrionalis*) captured in Nova Scotia between 2000 and 2019. Each boxplot is comprised of 35 points, each representing the estimated daily change in body mass for the group between Julian dates 227 and 262 (approximately August 15–September 19). Error bars represent the standard error of the estimated mean daily change in body mass.

The 15 fall groups (Figure 4; Tables 3 and 4) were assorted into three DTW clusters, which were largely distinguishable according to species (Figure 5). Differences by age class were less evident in northern myotis than little brown myotis, and juvenile little brown myotis were more closely associated with adult northern myotis than conspecific adults or juvenile northern myotis. The greatest differentiation was found among the two species‐associated clusters (cophenetic distance = 208.50). With the exception of juvenile northern myotis (*t*‐test, *p* = .01), the fall estimated rates of mass gain were generally similar among sexes in both species and were less variable in juveniles than adults (Levene's test, *p* < .001 for both species) (Figure 6). The estimated onsets of mass gain took place between Julian Day 227 and 255 (Table 4). Between the two species, rapid mass gain generally began earlier in little brown myotis and in adults of both species, but the age‐associated pattern was less distinct.

**TABLE 3:** Sampling characteristics of groups of little brown myotis (*Myotis lucifugus*) and northern myotis (*M. septentrionalis*) captured in Nova Scotia during the fall between 2009 and 2011.

Species	Sex	Year	Age	Earliest capture	Latest capture	Largest gap	*n*	Nights	Sites
*M. lucifugus*	Female	2009	Adult	227	274	4	162	25	6
*M. lucifugus*	Male	2009	Adult	227	279	5	242	27	6
*M. lucifugus*	Male	2009	Juvenile	227	279	12	49	17	6
*M. lucifugus*	Female	2010	Adult	226	273	10	97	22	9
*M. lucifugus*	Male	2010	Adult	209	276	17	189	29	12
*M. lucifugus*	Female	2011	Adult	223	269	8	65	12	6
*M. lucifugus*	Male	2011	Adult	223	269	8	167	16	6
*M. lucifugus*	Female	2011	Juvenile	198	269	15	48	14	11
*M. lucifugus*	Male	2011	Juvenile	198	269	12	71	15	10
*M. septentrionalis*	Female	2009	Adult	227	267	12	87	19	6
*M. septentrionalis*	Male	2009	Adult	227	274	7	129	21	6
*M. septentrionalis*	Female	2009	Juvenile	227	265	7	41	16	6
*M. septentrionalis*	Male	2009	Juvenile	227	274	9	67	17	6
*M. septentrionalis*	Female	2010	Adult	226	262	10	70	21	11
*M. septentrionalis*	Male	2010	Adult	209	276	17	130	26	13

*Note*: Earliest capture and latest capture denote the date (Julian date) of the first and last capture in each group, and the largest gap in sampling within those dates is largest gap. The sample size, number of unique capture nights, and total number of capture sites are given as *n*, nights, and sites, respectively.

**TABLE 4:** Summary of the LOESS‐fitted interpolations of mass values of groups of little brown myotis (*Myotis lucifugus*) and northern myotis (*M. septentrionalis*) captured during the fall in Nova Scotia between 2009 and 2011.

Species	Sex	Year	Age	*SE*	Earliest mass (g)	Latest mass (g)	Max change (g)	Onset
*M. lucifugus*	Female	2009	Adult	1.00	7.67	9.28	2.10	244
*M. lucifugus*	Male	2009	Adult	1.21	7.20	9.07	1.92	229
*M. lucifugus*	Male	2009	Juvenile	0.77	6.67	6.89	0.46	241
*M. lucifugus*	Female	2010	Adult	1.02	7.15	9.23	2.44	239
*M. lucifugus*	Male	2010	Adult	1.03	7.45	8.80	1.69	227
*M. lucifugus*	Female	2011	Adult	0.94	7.56	9.04	1.53	249
*M. lucifugus*	Male	2011	Adult	1.10	7.41	9.51	2.20	238
*M. lucifugus*	Female	2011	Juvenile	0.64	6.65	7.89	1.24	227
*M. lucifugus*	Male	2011	Juvenile	0.77	6.62	8.04	1.41	249
*M. septentrionalis*	Female	2009	Adult	0.52	6.73	7.32	1.38	248
*M. septentrionalis*	Male	2009	Adult	0.71	6.75	7.07	1.00	248
*M. septentrionalis*	Female	2009	Juvenile	0.89	6.11	6.57	0.73	245
*M. septentrionalis*	Male	2009	Juvenile	0.61	6.13	5.86	0.48	255
*M. septentrionalis*	Female	2010	Adult	0.91	6.42	7.84	1.84	249
*M. septentrionalis*	Male	2010	Adult	0.63	6.42	6.54	0.69	251

*Note*: Earliest mass and latest mass describe the fitted mass value on days‐of‐year 227 and 262, respectively, and max change denotes the largest net difference present between any two fitted values in the sampling period. Standard error (*SE*) describes the error of each full LOESS fit (span = 0.75), including all values between the earliest available capture until the latest available capture.

**FIGURE 7:**
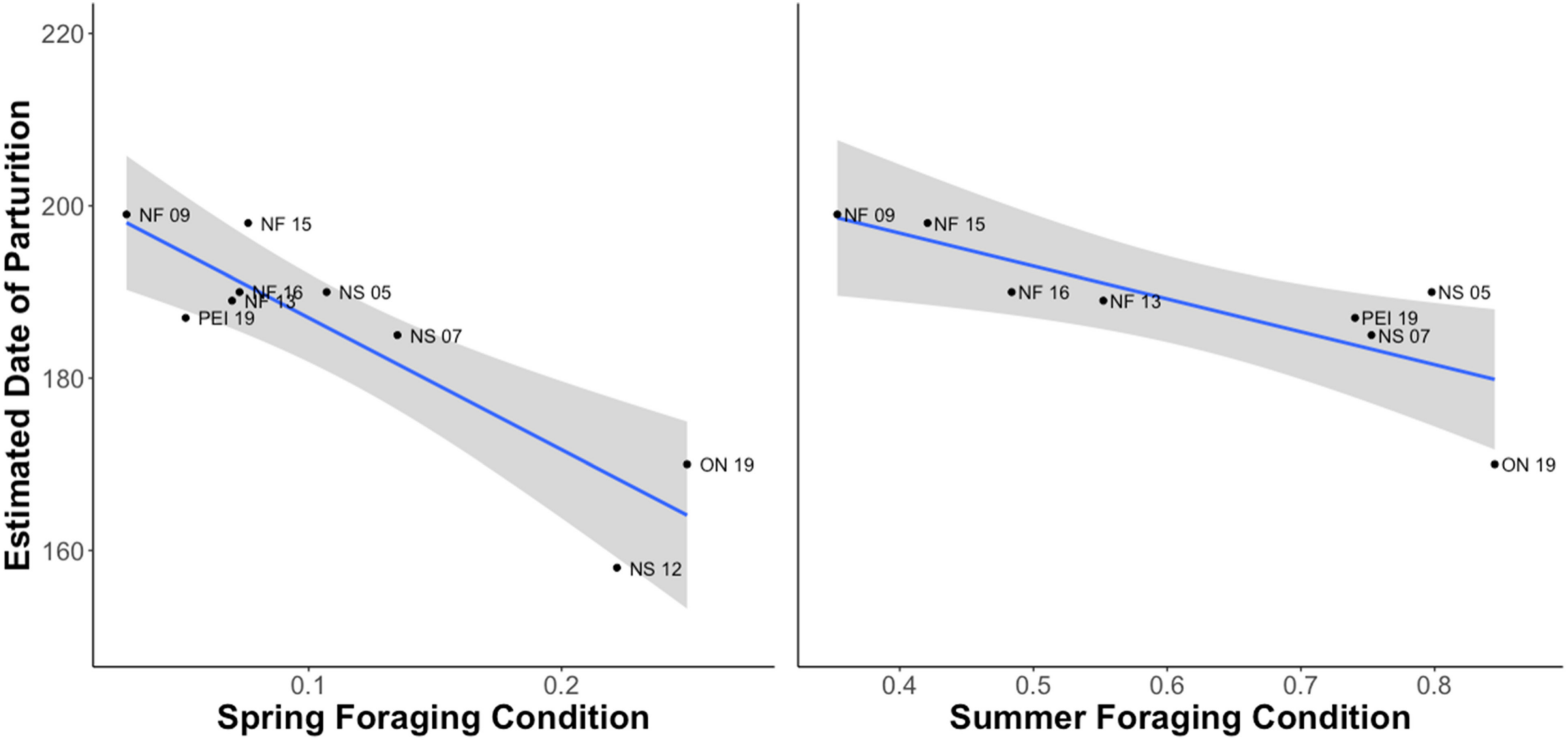
Linear relationship between spring and summer foraging conditions and the estimated date of parturition in little brown myotis (*Myotis lucifugus*) and northern myotis (*M. septentrionalis*) captured in New Brunswick (NB), Nova Scotia (NS), Prince Edward Island (PEI), Newfoundland (NF), and Ontario (ON) between 2005 and 2019. Each point represents the estimated date of parturition for one group, whose year of capture is indicated with its province abbreviation.

There was a significant relationship between the estimated date of parturition and spring foraging condition (SpSFC: *R* ^2^ = .792, Pearson: *p* = .001, *df* = 7), but not with summer foraging condition (SuSFC: *R* ^2^ = .643, Pearson: *p* = .017, *df* = 7) (Figure 7). The summer foraging condition estimate from Nova Scotia in 2012 was an outlier (Cook's distance = 0.465), and was thus excluded from the summer analysis.

## 4 DISCUSSION

The summer body mass variation patterns of adult male and female little brown myotis and northern myotis were both visually and statistically distinguishable. Indeed, female patterns contained mid‐season parturition peaks and were more variable than those of males. It is unsurprising that female little brown myotis were the most differentiated from the other groups, given both their peaks and their relatively greater mass than northern myotis (Jung et al., 2006; Kunz et al., 1998; Kurta et al., 1989; Reynolds & Kunz, 2000; Van Zyll De Jong, 1985). Furthermore, the low and consistent early summer mass values for male groups suggest that the early summer period is characterized by some combination of late emergence (Czenze & Willis, 2015) and small net positive energy budget balances. Similarly, the variance in early summer female mass supports the contention that female emergence times may vary according to factors like body condition, reproductive status, or weather which may differ among groups (Frick, Reynolds, et al., 2010; Jonasson & Willis, 2011).

The fall clustering outcomes indicate lower behavioral differentiation relative to summer and did not provide evidence for differentiation according to sex. There was weak visual evidence for delayed rapid mass gain in juveniles, whose onset estimates were generally similar to adults in the same year. Temporal asynchrony in rapid mass gain was evident in Kunz et al. (1998), but McGuire et al. (2009) observed no clear period of juvenile rapid mass gain during their study. In light of these studies, our clustering and onset estimates suggest that if juveniles achieve and sustain high rates of mass gain like adults, it occurs later in Nova Scotia than could be observed within the sampling period. Despite an upward bend in many of the fitted juvenile lines, the relatively low variation and mean in mass gain rates in juveniles suggest that their prehibernation strategy may be better characterized as a single long effort following weaning. Conversely, we found clear evidence that adults undergo a transition from summer behavior to a period of rapid mass gain characterized by rates as high as 0.1 g/day (Figure 6), like those observed in Kunz et al. (1998). Regardless of which factors drive this difference, our work provides further support for the existence of age‐dependent prehibernation strategies in both little brown myotis and northern myotis.

One contributing factor to the timing of age‐ or sex‐specific strategies by individuals is the weather. In adult female bats, we found that the association of weather with the estimated date of parturition was especially strong in spring. The relatively greater importance of spring weather over summer is intuitive, considering that gestation begins very shortly after emergence (O'Farrell & Studier, 1973; Wimsatt, 1945), and that favorable springs should facilitate earlier parturition (Linton & Macdonald, 2018), regardless of the quality of the subsequent summer. One proposed mechanism for this relationship is non‐random pre‐emergence arousal patterns in little brown myotis, which suggest that females in good body condition capitalize on rare warm early‐spring nights (Czenze et al., 2017; Czenze & Willis, 2015). Years with more favorable nights early in the spring may provide benefits to emergent females in a wider range of body conditions, and therefore move the mean parturition date up. Alternatively, spring conditions may simply dictate the timing of emergence and thus the onset of gestation and associated rapid mass gain. Although Czenze and Willis (2015) demonstrated that emergence timing at a hibernaculum in Manitoba was associated with barometric pressure changes, Meyer et al. (2016) found no such relationship at a hibernaculum further South in Wisconsin. In context with the known influence of body mass on emergence timing (Czenze & Willis, 2015; Frick, Reynolds, et al., 2010; Norquay & Willis, 2014), it is, therefore, less likely that foraging conditions drove early emergence, but instead facilitated gestation for those females that had already emerged.

In summer sampling, all‐male groups demonstrated consistently positive and less variable rates of daily mass change than female groups, but it should be noted that the mid‐summer negative slopes associated with female groups can generally be attributed to the effect of parturition on a group's mean mass. Regardless, female groups demonstrated higher rates of mass gain than concurrent male groups, especially during early summer. This disparity indicates that if pregnancy is facilitated by favorable foraging conditions (Anthony & Kunz, 1977; Arlettaz et al., 2001), male body mass is not affected to the same degree, likely due to the lack of pressure to develop significant sperm stores so early in the season. The presence of this pattern in male little brown myotis and northern myotis groups across the years supports the contention that male temperate bats minimize their net energy budget throughout the summer (Barclay, 1991; Wilkinson & Barclay, 1997) due to the higher costs of flight at greater body masses (Winter & Von Helversen, 1998). Complementing further mass variation analysis with temperature‐sensitive radio‐telemetry data (e.g., Barclay et al., 1996) may clarify whether these patterns are driven by lower rates of foraging (Barclay, 1991), increased use of torpor (Kurta & Kunz, 1988), or less efficient foraging (Wilkinson & Barclay, 1997). In particular, such analysis would identify the relationships between nightly foraging frequency or duration and daily patterns of thermoregulation.

There are some important considerations to these results. First, the variation in a LOESS‐fitted line is a function of the chosen span parameter (Cleveland & Devlin, 1988). Because we selected a conservative span parameter (0.75), we conceded that the fitted lines would not capture short term changes in average mass. Similarly, derivatives calculated for each group also reflect the variability introduced by the span parameter and thus do not reliably capture short‐term variation. For this reason, we do not consider the short‐term fluctuations in fall fits to be informative, but instead used the distribution of derivatives to contextualize our conclusions. Additionally, group composition accounted for sex, year, and province, but included captures pooled from sites across each province, which may have masked intra‐site variation. We recognize both sampling frequency and site distribution as major limitations in analyses of historical datasets like this one and emphasize the value of ongoing long‐term monitoring.

One important consideration for our results was the timeline of our data collection relative to the introduction and impact of WNS in our study region. After its detection in 2006 (Blehert et al., 2009), WNS spread to Atlantic Canada by the early 2010s and has since driven population declines across Eastern Canada (Balzer et al., 2021; COSEWIC 2013). Although most of our data predate WNS, we recognize the potential for spurious conclusions due to the absence of post‐WNS northern myotis and the inclusion of post‐WNS little brown myotis (e.g., PEI 2019). One of the most notable potential influences of WNS on our study was the absence of northern myotis after 2010. They have likely undergone a much more drastic decline in Eastern Canada relative to little brown myotis (Balzer et al., 2021), which would explain insufficient capture records for their post‐WNS inclusion in our work. Another possible influence of WNS could be the selection for certain mass gain strategies. Indeed, Haase et al. (2021) indicated that mass is an important predictor of overwinter survival, which may reflect a selection pressure to enter hibernation at a critical threshold of increased body mass. If that pressure exists, the large differentiation among the female little brown myotis and other summer groups (Figure 1) may in part be explained by ongoing selection for a greater mass strategy.

Given the likelihood that greater fat reserves are associated with increased WNS survival (Cheng et al., 2019; Lacki et al., 2015), our results may provide some insight into the vulnerability of certain demographics of bats to WNS. Our work emphasizes the disparity in fall mass between adults and juveniles within our study species. If this disparity is also reflected in a lower juvenile percent body fat upon entering hibernation (Kunz et al., 1998), WNS may pose a greater immediate survival risk to juveniles than adults. Our results also provide further support for the link between foraging conditions and the phenology of energy‐intensive behaviors, such as reproduction. In years of poor spring and summer foraging conditions, delayed parturition shortens the period for juveniles to gain mass prior to hibernation, which may limit their ability to achieve sufficient overwintering mass under WNS pressure. Differences in foraging capacity (Anthony & Kunz, 1977) during poor fall conditions may further compound this challenge for juveniles. As WNS continues to affect populations across North America, the adaptive capacity of populations to its impact will further clarify the degree to which these and other factors influence WNS resistance (Auteri & Knowles, 2020).

Collectively, each objective of this project supports the hypothesis that sex‐ and age‐specific energy allocation regimes of little brown myotis and northern myotis confer similarly disparate patterns of body mass variation. In particular, our results illustrate the drastic body mass changes in females in early summer, and the concurrent low rates of body mass change in males. Furthermore, we demonstrated that weather conditions are important for the reproductive success of female temperate hibernating bats. Fall analysis identified the presence of disparate prehibernation strategies among adults and juveniles. Both the summer and fall patterns were identifiable through time‐series clustering, and demonstrate its use as a viable technique for characterizing body mass patterns among populations. In tandem with time‐series modeling, it should be considered a viable tool to identify phenological patterns in populations with important annual behaviors (e.g., molting birds: Sjöberg, 1988).

## AUTHOR CONTRIBUTIONS

**Evan W. Balzer:** Conceptualization (lead); data curation (equal); formal analysis (lead); funding acquisition (supporting); investigation (equal); methodology (lead); project administration (equal); resources (supporting); writing – original draft (lead); writing – review and editing (equal). **Adam D. Grottoli:** Conceptualization (supporting); data curation (equal); formal analysis (supporting); methodology (equal); project administration (supporting); writing – original draft (supporting); writing – review and editing (equal). **Lynne E. Burns:** Conceptualization (supporting); data curation (equal); resources (supporting); writing – original draft (supporting); writing – review and editing (supporting). **Hugh G. Broders:** Conceptualization (supporting); data curation (equal); formal analysis (supporting); funding acquisition (lead); investigation (equal); methodology (supporting); project administration (equal); resources (lead); supervision (lead); writing – original draft (supporting); writing – review and editing (equal).

## CONFLICTS OF INTEREST

We have no conflicts of interest to declare.

## Data Availability

Data are available on Dryad (https://doi.org/10.5061/dryad.pc866t1rq), with capture site latitude and longitude redacted due to the endangered status of the study species.

## References

[ece39230-bib-0001] Aghabozorgi, S. , Seyed Shirkhorshidi, A. , & Ying Wah, T. (2015). Time‐series clustering ‐ a decade review. Information Systems, 53, 16–38.

[ece39230-bib-0002] Amin, T. B. , & Mahmood, I. (2008). Speech recognition using dynamic time warping. 2nd International Conference on Advances in Space Technologies, 2, 74–79.

[ece39230-bib-0003] Anthony, E. L. P. , & Kunz, T. H. (1977). Feeding strategies of the little brown bat, *Myotis lucifugus*, in southern New Hampshire. Ecology, 58, 775–786.

[ece39230-bib-0004] Anthony, E. L. P. , Stack, M. H. , & Kunz, T. H. (1981). Night roosting and the nocturnal time budget of the little brown bat, *Myotis lucifugus*: Effects of reproductive status, prey density, and environmental conditions. Oecologia, 51, 151–156. 28310074 10.1007/BF00540593

[ece39230-bib-0005] Arlettaz, R. , Christe, P. , Lugon, A. , Perrin, N. , & Vogel, P. (2001). Food availability dictates the timing of parturition in insectivorous mouse‐eared bats. Oikos, 95, 105–111.

[ece39230-bib-0006] Auteri, G. G. , & Knowles, L. L. (2020). Decimated little brown bats show potential for adaptive change. Scientific Reports, 10, 1–10. 32080246 10.1038/s41598-020-59797-4 PMC7033193

[ece39230-bib-0007] Balzer, E. W. , Grottoli, A. D. , Phinney, L. J. , Burns, L. E. , Vanderwolf, K. J. , & Broders, H. G. (2021). Capture rate declines of northern myotis in the Canadian Maritimes. Wildlife Society Bulletin, 45, 719–724.

[ece39230-bib-0008] Barclay, R. M. R. (1991). Population structure of temperate zone insectivorous bats in relation to foraging behaviour and energy demand. Journal of Animal Ecology, 60, 165–178.

[ece39230-bib-0009] Barclay, R. M. R. , Kalcounis, M. C. , Crampton, L. H. , Stefan, C. , Vonhof, M. J. , Wilkinson, L. , & Brigham, R. M. (1996). Can external Radiotransmitters be used to assess body temperature and torpor in bats? Journal of Mammalogy, 77, 1102–1106.

[ece39230-bib-0010] Berndt, D. , & Clifford, J. (1994). Using dynamic time warping to find patterns in time series. Workshop on Knowledge Discovery in Databases, 398, 359–370.

[ece39230-bib-0011] Besler, N. K. , & Broders, H. G. (2019). Combinations of reproductive, individual, and weather effects best explain torpor patterns among female little brown bats (*Myotis lucifugus*). Ecology and Evolution, 9, 5158–5171. 31110669 10.1002/ece3.5091 PMC6509385

[ece39230-bib-0012] Blehert, D. S. , Hicks, A. C. , Behr, M. , Meteyer, C. U. , Berlowski‐zier, B. M. , Buckles, E. L. , Coleman, J. T. H. , Darling, S. R. , Gargas, A. , Niver, R. , Okoniewski, J. C. , Rudd, R. J. , & Stone, W. B. (2009). Bat white‐nose syndrome: An emerging fungal pathogen? Science, 323, 227. 18974316 10.1126/science.1163874

[ece39230-bib-0013] Bonan, G. (2002). Ecological climatology (1st ed.). Cambridge University Press.

[ece39230-bib-0014] Burles, D. W. , Brigham, R. M. , Ring, R. A. , & Reimchen, T. E. (2009). Influence of weather on two insectivorous bats in a temperate Pacific northwest rainforest. Canadian Journal of Zoology, 87, 132–138.

[ece39230-bib-0015] Burns, L. E. , & Broders, H. G. (2015). Who swarms with whom? Group dynamics of myotis bats during autumn swarming. Behavioral Ecology, 26, 866–876.

[ece39230-bib-0016] Caceres, M. C. , & Barclay, R. R. (2000). Myotis septentrionalis. Mammalian species 1–4 in Canada. Species at risk act recovery strategy series. Environment Canada.

[ece39230-bib-0017] Carey, H. V. , Andrews, M. T. , & Martin, S. L. (2003). Mammalian hibernation: Cellular and molecular responses to depressed metabolism and low temperature. Physiological Reviews, 83, 1153–1181. 14506303 10.1152/physrev.00008.2003

[ece39230-bib-0018] Chen, Y. , Kopp, G. A. , & Surry, D. (2002). Interpolation of wind‐induced pressure time series with an artificial neural network. Journal of Wind Engineering and Industrial Aerodynamics, 90, 589–615.

[ece39230-bib-0019] Cheng, T. L. , Gerson, A. , Moore, M. S. , Reichard, J. D. , DeSimone, J. , Willis, C. K. R. , Frick, W. F. , & Kilpatrick, A. M. (2019). Higher fat stores contribute to persistence of little brown bat populations with white‐nose syndrome. Journal of Animal Ecology, 88, 591–600. 30779125 10.1111/1365-2656.12954

[ece39230-bib-0020] Cheng, T. L. , Reichard, J. D. , Coleman, J. T. H. , Weller, T. J. , Thogmartin, W. E. , Reichert, B. E. , Bennett, A. B. , Broders, H. G. , Campbell, J. , Etchison, K. , Feller, D. J. , Geboy, R. , Hemberger, T. , Herzog, C. , Hicks, A. C. , Houghton, S. , Humber, J. , Kath, J. A. , King, R. A. , … Frick, W. F. (2021). The scope and severity of white‐nose syndrome on hibernating bats in North America. Conservation Biology, 35, 1586–1597. 33877716 10.1111/cobi.13739 PMC8518069

[ece39230-bib-0021] Ciechanowski, M. , Zaja̧c, T. , Biłas, A. , & Dunajski, R. (2007). Spatiotemporal variation in activity of bat species differing in hunting tactics: Effects of weather, moonlight, food abundance, and structural clutter. Canadian Journal of Zoology, 85, 1249–1263.

[ece39230-bib-0022] Cleveland, W. S. (1979). Robust locally weighted regression and smoothing scatterplots. Journal of the American Statistical Association, 74, 829–836.

[ece39230-bib-0023] Cleveland, W. S. , & Devlin, S. J. (1988). Locally weighted regression: An approach to regression analysis by local fitting. Journal of the American Statistical Association, 83, 596–610.

[ece39230-bib-0024] Committee on the Status of Endangered Wildlife in Canada (COSEWIC) . (2013). COSEWIC assessment and status report on the Little Brown Myotis Myotis lucifugus, Northern Myotis Myotis septentrionalis and Tri‐colored Bat Perimyotis subflavus in Canada. Committee on the Status of Endangered Wildlife in Canada. https://publications.gc.ca/site/eng/9.579593/publication.html

[ece39230-bib-0025] Czenze, Z. J. , Jonasson, K. A. , & Willis, C. K. R. (2017). Thrifty females, frisky males: Winter energetics of hibernating bats from a cold climate. Physiological and Biochemical Zoology, 90, 502–511. 28641050 10.1086/692623

[ece39230-bib-0026] Czenze, Z. J. , & Willis, C. K. R. (2015). Warming up and shipping out: Arousal and emergence timing in hibernating little brown bats (*Myotis lucifugus*). Journal of Comparative Physiology B: Biochemical, Systemic, and Environmental Physiology, 185, 575–586. 25809999 10.1007/s00360-015-0900-1

[ece39230-bib-0027] Davis, W. H. , & Hitchcock, H. (1965). Biology and migration of the bat, *Myotis lucifugus*, in New England. Journal of Mammalogy, 46, 296–313.

[ece39230-bib-0028] Dzal, Y. A. , & Brigham, R. M. (2013). The tradeoff between torpor use and reproduction in little brown bats (*Myotis lucifugus*). Journal of Comparative Physiology B: Biochemical, Systemic, and Environmental Physiology, 183, 279–288. 22972361 10.1007/s00360-012-0705-4

[ece39230-bib-0029] Environment and Climate Change Canada . (2018). Recovery strategy for little Brown myotis (Myotis lucifugus), Northerm myotis (Myotis septentrionalis), and tri‐Coloured bat (Perimyotis subflavus) in Canada. Species at risk act recovery strategy series. Environment Canada.

[ece39230-bib-0030] Fenton, B. (1970). Population studies of *Myotis lucifugus* (Chiroptera: Vespertilionidae) in Ontario. Life Sciences Contributions, Royal Ontario Museum, 77, 1–34.

[ece39230-bib-0031] Fenton, B. , & Barclay, R. (1980). Myotis lucifugus . American Society of Mammalogists, 142, 1–8.

[ece39230-bib-0032] Fenton, M. B. (1969). Summer activity of *Myotis lucifugus* (Chiroptera: Vespertilionidae) at hibernacula in Ontario and Quebec. Canadian Journal of Zoology, 47, 597–602.

[ece39230-bib-0033] Forrest, J. R. K. (2016). Complex responses of insect phenology to climate change. Current Opinion in Insect Science, 17, 49–54. 27720073 10.1016/j.cois.2016.07.002

[ece39230-bib-0034] Frick, W. F. , Pollock, J. F. , Hicks, A. C. , Langwig, K. E. , Reynolds, D. S. , Turner, G. G. , Butchkoski, C. M. , & Kunz, T. H. (2010). An emerging disease causes regional population collapse of a common north American bat species. Science, 329, 679–682. 20689016 10.1126/science.1188594

[ece39230-bib-0035] Frick, W. F. , Reynolds, D. S. , & Kunz, T. H. (2010). Influence of climate and reproductive timing on demography of little Brown myotis *Myotis lucifugus* . Journal of Animal Ecology, 79, 128–136. 19747346 10.1111/j.1365-2656.2009.01615.x

[ece39230-bib-0036] Geiser, F. (2004). Metabolic rate and body temperature reduction during hibernation and daily torpor. Annual Review of Physiology, 66, 239–274. 10.1146/annurev.physiol.66.032102.115105 14977403

[ece39230-bib-0037] Grindal, S. D. , Collard, T. S. , Brigham, R. M. , & Barclay, R. M. R. (1992). The influence of precipitation on reproduction by *Myotis* bats in British Columbia. The American Midland Naturalist, 128, 339–344.

[ece39230-bib-0038] Haase, C. G. , Fuller, N. W. , Dzal, Y. A. , Hranac, C. R. , Hayman, D. T. S. , Lausen, C. L. , Silas, K. A. , Olson, S. H. , & Plowright, R. K. (2021). Body mass and hibernation microclimate may predict bat susceptibility to white‐nose syndrome. Ecology and Evolution, 11, 506–515. 33437446 10.1002/ece3.7070 PMC7790633

[ece39230-bib-0039] Humphries, M. M. , Thomas, D. W. , & Kramer, D. L. (2003). The role of energy availability in mammalian hibernation: A cost–benefit approach. Physiological and Biochemical Zoology, 76, 165–179. 12794670 10.1086/367950

[ece39230-bib-0040] Jonasson, K. A. , & Willis, C. K. R. (2011). Changes in body condition of hibernating bats support the thrifty female hypothesis and predict consequences for populations with white‐nose syndrome. PLoS One, 6, 1–8. 10.1371/journal.pone.0021061 PMC3120823 21731647

[ece39230-bib-0041] Jung, T. S. , Slough, B. G. , Nagorsen, D. W. , Dewey, T. A. , & Powell, T. (2006). First records of the northern long‐eared bat, *Myotis septentrionalis*, in the Yukon territory. Canadian Field‐Naturalist, 120, 39–42.

[ece39230-bib-0042] Kronfeld‐Schor, N. , Richardson, C. , Silvia, B. A. , Kunz, T. H. , & Widmaier, E. P. (2000). Dissociation of leptin secretion and adiposity during prehibernatory fattening in little brown bats. American Journal of Physiology ‐ Regulatory Integrative and Comparative Physiology, 279, 1277–1281. 10.1152/ajpregu.2000.279.4.R1277 11003993

[ece39230-bib-0043] Kunz, T. H. , & Anthony, E. L. P. (1982). Age estimation and post‐natal growth in the bat *Myotis lucifugus* . Journal of Mammalogy, 63, 23–32.

[ece39230-bib-0044] Kunz, T. H. , Wrazen, J. A. , & Burnett, C. D. (1998). Changes in body mass and fat reserves in pre‐hibernating little brown bats (*Myotis lucifugus*). Ecoscience, 5, 8–17.

[ece39230-bib-0045] Kurta, A. , Bell, G. P. , Nagy, K. A. , & Kunz, T. H. (1989). Energetics of pregnancy and lactation in free‐ranging little brown bats (*Myotis lucifugus*). Physiological Zoology, 62, 804–818.

[ece39230-bib-0046] Kurta, A. , & Kunz, T. H. (1988). Roosting metabolic rate and body temperature of male little brown bats (*Myotis lucifugus*) in summer. Journal of Mammalogy, 69, 645–651.

[ece39230-bib-0047] Lacki, M. J. , Dodd, L. E. , Toomey, R. S. , Thomas, S. C. , Couch, Z. L. , & Nichols, B. S. (2015). Temporal changes in body mass and body condition of cave‐hibernating bats during staging and swarming. Journal of Fish and Wildlife Management, 6, 360–370.

[ece39230-bib-0048] Lane, J. E. , Kruuk, L. E. B. , Charmantier, A. , Murie, J. O. , & Dobson, F. S. (2012). Delayed phenology and reduced fitness associated with climate change in a wild hibernator. Nature, 489, 554–557. 22878721 10.1038/nature11335

[ece39230-bib-0049] Lepot, M. , Aubin, J. B. , & Clemens, F. H. L. R. (2017). Interpolation in time series: An introductive overview of existing methods, their performance criteria and uncertainty assessment. Water, 9(796), 1–20.29225961

[ece39230-bib-0050] Lilley, T. M. , Johnson, J. S. , Ruokolainen, L. , Rogers, E. J. , Wilson, C. A. , Schell, S. M. , Field, K. A. , & Reeder, D. M. (2016). White‐nose syndrome survivors do not exhibit frequent arousals associated with *Pseudogymnoascus destructans* infection. Frontiers in Zoology, 13, 1–8. 26949407 10.1186/s12983-016-0143-3 PMC4778317

[ece39230-bib-0051] Linton, D. M. , & Macdonald, D. W. (2018). Spring weather conditions influence breeding phenology and reproductive success in sympatric bat populations. Journal of Animal Ecology, 87, 1080–1090. 29635800 10.1111/1365-2656.12832

[ece39230-bib-0052] Linton, D. M. , & Macdonald, D. W. (2019). Roost composition and sexual segregation in a lowland population of Daubenton's bats (*Myotis daubentonii*). Acta Chiropterologica, 21, 129–137.

[ece39230-bib-0053] Linton, D. M. , & Macdonald, D. W. (2020). Phenology of reproductive condition varies with age and spring weather conditions in male *Myotis daubentonii* and *M. nattereri* (Chiroptera: Vespertilionidae). Scientific Reports, 10, 1–10. 32313091 10.1038/s41598-020-63538-y PMC7171103

[ece39230-bib-0054] McGuire, L. P. , Fenton, M. B. , & Guglielmo, C. G. (2009). Effect of age on energy storage during prehibernation swarming in little brown bats (*Myotis lucifugus*). Canadian Journal of Zoology, 87, 515–519.

[ece39230-bib-0055] McGuire, L. P. , Muise, K. A. , Shrivastav, A. , & Willis, C. K. R. (2016). No evidence of hyperphagia during pre‐hibernation in a northern population of little brown bats (*Myotis lucifugus*). Canadian Journal of Zoology, 94, 821–827.

[ece39230-bib-0056] Meyer, G. A. , Senulis, J. A. , & Reinartz, J. A. (2016). Effects of temperature and availability of insect prey on bat emergence from hibernation in spring. Journal of Mammalogy, 97, 1623–1633.

[ece39230-bib-0057] Nelson, R. A. , Folk, G. E. , Pfeiffer, E. W. , Craighead, J. J. , Jonkel, C. J. , & Steiger, D. L. (1983). Behavior, biochemistry, and hibernation in black, grizzly, and polar bears. International Conference on Bear Research and Management, 5, 284–290.

[ece39230-bib-0058] Newton, I. , & Dale, L. C. (1996). Bird migration at different latitudes in eastern North America. The Auk, 113, 626–635.

[ece39230-bib-0059] Norquay, K. J. O. , & Willis, C. K. R. (2014). Hibernation phenology of *Myotis lucifugus* . Journal of Zoology, 294, 85–92.

[ece39230-bib-0060] O'Farrell, M. , & Studier, E. (1973). Reproduction, growth, and development in *Myotis thysanodes* and *M. lucifugus* (Chiroptera: Vespertilionidae). Ecology, 54, 18–30.

[ece39230-bib-0061] Parsons, K. N. , Jones, G. , & Greenaway, F. (2003). Swarming activity of temperate zone microchiropteran bats: Effects of season, time of night and weather conditions. Journal of Zoology, 261, 257–264.

[ece39230-bib-0062] Pigliucci, M. (2001). Phenotypic plasticity: Beyond nature and nurture. Johns Hopkins University Press.

[ece39230-bib-0063] Rabiner, L. , Rosenberg, A. , & Levinson, S. (1978). Considerations in dynamic time warping algorithms for discrete word recognition. IEEE Transactions on Acoustics, Speech and Signal Processing, 26, 575–582.

[ece39230-bib-0064] Racey, P. A. , & Swift, S. M. (1981). Variation in gestation length in a Colony of pipistrelle bats (*Pipistrellus pipistrellus*) from year to year. Journal of Reproduction and Fertility, 61, 123–129. 7452610 10.1530/jrf.0.0610123

[ece39230-bib-0065] Reeder, D. A. M. , Frank, C. L. , Turner, G. G. , Meteyer, C. U. , Kurta, A. , Britzke, E. R. , Vodzak, M. E. , Darling, S. R. , Stihler, C. W. , Hicks, A. C. , Jacob, R. , Grieneisen, L. E. , Brownlee, S. A. , Muller, L. K. , & Blehert, D. S. (2012). Frequent arousal from hibernation linked to severity of infection and mortality in bats with white‐nose syndrome. PLoS One, 7, 1–10. 10.1371/journal.pone.0038920 PMC3380050 22745688

[ece39230-bib-0066] Reynolds, S. , & Kunz, T. (2000). Changes in body composition during reproduction and postnatal growth in the little brown bat, *Myotis lucifugus* (Chiroptera: Vespertilionidae). Ecoscience, 7, 10–17.

[ece39230-bib-0067] Rodrigues, L. , Zahn, A. , Rainho, A. , & Palmeirim, J. M. (2003). Contrasting the roosting behaviour and phenology of an insectivorous bat (*Myotis myotis)* in its southern and northern distribution ranges. Mammalia, 67, 321–335.

[ece39230-bib-0068] Rughetti, M. , & Toffoli, R. (2014). Sex‐specific seasonal change in body mass in two species of Vespertilionid bats. Acta Chiropterologica, 16, 149–155.

[ece39230-bib-0069] Sakoe, H. , & Chiba, S. (1971). A dynamic programming approach to continuous speech recognition. Proceedings of the Seventh International Congress on Acoustics, 3, 65–69.

[ece39230-bib-0070] Sardá‐Espinosa, A. (2019). Time‐series clustering in R using the Dtwclust package. R Journal, 11, 1–22.

[ece39230-bib-0071] Schowalter, D. B. (1980). Swarming, reproduction, and early hibernation of *Myotis lucifugus* and *M. volans* in Alberta, Canada. Journal of Mammalogy, 61, 350–354.

[ece39230-bib-0072] Sjöberg, K. (1988). The flightless period of free‐living male teal *Anas crecca* in northern Sweden. Ibis, 130, 164–171.

[ece39230-bib-0073] Snell‐Rood, E. C. (2013). An overview of the evolutionary causes and consequences of behavioural plasticity. Animal Behaviour, 85, 1004–1011.

[ece39230-bib-0074] Speakman, J. R. , & Rowland, A. (1999). Preparing for inactivity: How insectivorous bats deposit a fat store for hibernation. Proceedings of the Nutrition Society, 58, 123–131. 10343349 10.1079/pns19990017

[ece39230-bib-0075] Stawski, C. , Willis, C. K. R. , & Geiser, F. (2014). The importance of temporal heterothermy in bats. Journal of Zoology, 292, 86–100.

[ece39230-bib-0076] Stearns, S. C. (1976). Life‐history tactics: A review of the ideas. The Quarterly Review of Biology, 51, 3–47. 778893 10.1086/409052

[ece39230-bib-0077] Sunday, J. M. , Bates, A. E. , & Dulvy, N. K. (2011). Global analysis of thermal tolerance and latitude in ectotherms. Proceedings of the Royal Society B: Biological Sciences, 278, 1823–1830. 10.1098/rspb.2010.1295 PMC3097822 21106582

[ece39230-bib-0078] Thieurmel, B. , & Elmarhraoui, A. (2019). *Suncalc: Compute sun position, sunlight phases, moon position and lunar phase*. Version 0.5.0.

[ece39230-bib-0079] Thomas, D. W. , Dorais, M. , & Bergeron, J.‐M. (1990). Winter energy budgets and cost of arousals for hibernating little brown bats, *Myotis lucifugus* . Journal of Mammalogy, 71, 475–479.

[ece39230-bib-0080] Thomas, D. W. , Fenton, B. M. , & Barclay, R. M. R. (1979). Social behavior of the little brown bat, *Myotis lucifugus*: I. mating behavior. Behavioural Ecology and Sociobiology, 6, 129–136.

[ece39230-bib-0081] Townsend, K. L. , Kunz, T. H. , & Widmaier, E. P. (2008). Changes in body mass, serum leptin, and mRNA levels of leptin receptor isoforms during the premigratory period in *Myotis lucifugus* . Journal of Comparative Physiology B: Biochemical, Systemic, and Environmental Physiology, 178, 217–223. 17962952 10.1007/s00360-007-0215-y

[ece39230-bib-0082] Van Zyll De Jong, C. G. (1985). Handbook of Canadian mammals: Bats (2nd ed.). National Museums of Canada, Ottawa.

[ece39230-bib-0083] Ward, J. H. (1963). Hierarchical grouping to optimize an objective function. Journal of the American Statistical Association, 58, 236–244.

[ece39230-bib-0084] Whitaker, J. O. , & Gummer, S. L. (1992). Hibernation of the big brown bat, *Eptesicus fuscus*, in buildings. Journal of Mammalogy, 73, 312–316.

[ece39230-bib-0085] Wilkinson, L. C. , & Barclay, R. M. R. (1997). Differences in the foraging behaviour of male and female big brown bats (*Eptesicus fuscus*) during the reproductive period. Ecoscience, 4, 279–285.

[ece39230-bib-0086] Willis, C. K. R. , Brigham, R. M. , & Geiser, F. (2006). Deep, prolonged torpor by pregnant, free‐ranging bats. Naturwissenschaften, 93, 80–83. 16456644 10.1007/s00114-005-0063-0

[ece39230-bib-0087] Wimsatt, W. A. (1945). Notes on breeding behavior, pregnancy, and parturition in some Vespertilionid bats of the eastern United States. Journal of Mammalogy, 26, 23–33.

[ece39230-bib-0088] Wimsatt, W. A. (1960). An analysis of parturition in Chiroptera, including new observations on *Myotis lucifugus* . Journal of Mammalogy, 41, 183–200.

[ece39230-bib-0089] Winter, Y. , & Von Helversen, O. (1998). The energy cost of flight: Do small bats Fly more cheaply than birds? Journal of Comparative Physiology ‐ B Biochemical, Systemic, and Environmental Physiology, 168, 105–111. 9542147 10.1007/s003600050126

